# Host–Parasite Interactions in Human Malaria: Clinical Implications of Basic Research

**DOI:** 10.3389/fmicb.2017.00889

**Published:** 2017-05-18

**Authors:** Pragyan Acharya, Manika Garg, Praveen Kumar, Akshay Munjal, K. D. Raja

**Affiliations:** ^1^Department of Biochemistry, All India Institute of Medical SciencesNew Delhi, India; ^2^Department of Biochemistry, Jamia Hamdard UniversityNew Delhi, India

**Keywords:** *Plasmodium*, malaria, host–parasite interaction, protein, cytokines, invasion, direct interaction, indirect interaction

## Abstract

The malaria parasite, *Plasmodium*, is one of the oldest parasites documented to infect humans and has proven particularly hard to eradicate. One of the major hurdles in designing an effective subunit vaccine against the malaria parasite is the insufficient understanding of host–parasite interactions within the human host during infections. The success of the parasite lies in its ability to evade the human immune system and recruit host responses as physiological cues to regulate its life cycle, leading to rapid acclimatization of the parasite to its immediate host environment. Hence understanding the environmental niche of the parasite is crucial in developing strategies to combat this deadly infectious disease. It has been increasingly recognized that interactions between parasite proteins and host factors are essential to establishing infection and virulence at every stage of the parasite life cycle. This review reassesses all of these interactions and discusses their clinical importance in designing therapeutic approaches such as design of novel vaccines. The interactions have been followed from the initial stages of introduction of the parasite under the human dermis until asexual and sexual blood stages which are essential for transmission of malaria. We further classify the interactions as “direct” or “indirect” depending upon their demonstrated ability to mediate direct physical interactions of the parasite with host factors or their indirect manipulation of the host immune system since both forms of interactions are known to have a crucial role during infections. We also discuss the many ways in which this understanding has been taken to the field and the success of these strategies in controlling human malaria.

## Introduction

Malaria is one of the oldest documented human diseases, yet it is one of the most prevalent human infectious diseases even today. According to the latest estimates from WHO, there were 214 million new malaria cases and an estimated 4, 38,000 malaria deaths in 2015 (World Health Organization, 2015). Malaria is caused by protozoan parasites of the genus *Plasmodium*. *Plasmodium falciparum* is the most virulent form known among the five species (*P. vivax, P. ovale, P. malariae*, and *P. knowlesi*) that infect human beings (Sultan, 1999).

Human infection by the malaria parasite, specifically *P. falciparum*, can result in three different manifestations- asymptomatic malaria, mild (or uncomplicated) malaria, and severe (or complicated) malaria (World Health Organization, 2014; Chen et al., 2016). Asymptomatic malaria is defined as the presence of parasites in peripheral circulation in the absence of any of the symptoms that are associated with malaria infections such as fever and chills, in the absence of any antimalarial treatment (Chen et al., 2016). This is thought to result from partial immunity that may control, but not eliminate infection. Mild or uncomplicated malaria is when malaria infection is accompanied by fever and chills, and mild acute symptoms accompanied by parasitemia in peripheral blood smears. Severe malaria occurs when the acute symptoms associated with malaria infections increase in severity and affect the functioning of several organs, including the brain in cerebral malaria (Bartoloni and Zammarchi, 2012). WHO has a set of defined criteria that form the basis for the diagnosis of severe malaria (World Health Organization, 2014). Mortality during malaria infections is associated with severe disease. *P. falciparum* is typically associated with severe malaria, although *P. vivax* and *P. knowlesi* have been shown to associate with severe diseases less frequently. Although it is widely recognized that a multitude of factors influence disease outcomes in malaria, the identification of specific factors has been a slow process. We know today that parasite species and strain, host immune responses, host genetics, treatment regimen, all affect the outcome of malaria infections. It is important to review all the recent evidence in literature that facilitate our understanding of host–parasite interactions during human malaria infections, which will be crucial in understanding mechanisms of disease as well as discovering new targets for intervention. Indeed, some of the discussed interactions are already the focus of worldwide efforts toward the development of novel vaccines as well as drugs.

The life cycle of the malaria parasite is extremely complex and is shared between two hosts – human beings and the female *Anopheles* mosquito. The parasite enters the human host just below the skin and stays there for about 3 h (Ejigiri and Sinnis, 2009). The sporozoite form of the parasite first invades the liver hepatocytes, where it matures into merozoites that proceed to infect mature human erythrocytes. Within the erythrocyte, the parasite grows and multiplies in a cyclic fashion (Silvie et al., 2008). In each cycle of its growth within the erythrocyte, the parasite infects the erythrocyte as a merozoite, establishes a vacuole around itself as a ring stage, matures as a metabolically active trophozoite and replicates as a schizont. New infective merozoites are formed by mitosis in the schizont stages subsequent to which the RBC ruptures, releasing the daughter merozoites into circulation in order to establish fresh infection (Centers for Disease Control and Prevention, 2016). The rupture of the mature schizont is accompanied by the release of many intra-parasitic components that are known to elicit host inflammatory responses (Bousema et al., 2014; Centers for Disease Control and Prevention, 2016). Some of the parasites are programmed to develop into the sexual stage gametocytes that are picked up by the female *Anopheles* mosquito during itsblood meal. After 10–12 days of development in the mosquito midgut, they re-emerge as sporozoites in its salivary glands and re-enter human circulation upon fresh infection following mosquito bite (Dantzler et al., 2015; Josling and Llinas, 2015).

It is clear that parasite infection of the human liver, erythrocytes and the mosquito midgut are crucial to the establishment of infection and survival of the parasite within the host and all of these involve molecular interactions between the human host and the malaria parasite. *Plasmodium* spp. are known to modulate host immune responses using several strategies. In the following sections, we discuss these host–parasite interactions with a focus on those that have been used as candidates or targets in the development of vaccination strategies. We define “direct” interactions as those where a parasite-encoded factor has a demonstrated direct physical interaction with a host factor, effecting virulence, or disease outcomes in any way. We define “indirect” interactions as those where a parasite or host factor influences disease outcomes by modulating the host immune responses to parasite infections. These interactions are crucial to our understanding of malaria pathogenesis as well as toward identification of targets of therapeutic interventions. They are also fascinating examples of years of co-evolution between the malaria parasite and the human host. In endemic areas, malaria infections consist of infections of one the five species *P. falciparum, P. vivax, P. malariae, P. ovale*, and *P. knowlesi*, the first two being the most prevalent. Co-infections are also common in some areas. However, most of the discussion below focuses on *P. falciparum* malaria since most studies on human malaria has been carried out in this species. Malaria is an age-old disease and the malaria parasites, *P. vivax* in particular, is believed to have perfected the art of benign virulence and survival within human beings. However, recent reports of severe malaria during *P. vivax* infections have given rise to several questions about *P. vivax* interactions with its human host (Versiani et al., 2013). These questions are important areas of future research.

A majority of important host–parasite interaction data have been gleaned from elegant studies conducted in animal models of malaria and these have been comprehensively reviewed earlier (Craig et al., 2012). While animal models provide invaluable, reliable, reproducible, and manipulatable systems in which to study overall disease pathogenesis or carry out preliminary drug development studies, there have been reports of deviations from molecular details in human malaria infection (Craig et al., 2012). One of the key pathological features of human malaria is sequestration of parasites in specific tissues based on the specific binding phenotype of the parasite. One of the most severe consequences of this is human cerebral malaria that results from sequestration of infected RBCs (iRBCs) in the brain microvasculature (Craig et al., 2012). The rodent parasite *P. berghei* (ANKA) is used to simulate human cerebral malaria in rodents, resulting in experimental cerebral malaria (Craig et al., 2012). However, the mechanism of experimental cerebral malaria is still not well understood in terms of its molecular interaction and cytoadherence- the most important determinant of parasite binding phenotypes in human severe malaria. As a result, even though the rodent experimental cerebral malaria model has been useful to study host inflammatory responses to a virulent parasite strain, it cannot duplicate the molecular cocktail that results in human cerebral/severe malaria. In addition, host genetics, species and molecules are significantly different between humans and rodent models which have been the most popular model systems for the study of malaria pathogenesis, and intervention studies (Craig et al., 2012). Therefore, due to the above factors, and the prior presence of comprehensive literature on rodent malaria models, the present review focuses on human malaria as far as possible, and discusses their clinical implications.

## Direct Interactions

### Initial Interactions: the Skin and the Liver

The malaria parasites’ first encounter with human cells occurs in the human dermis where 20–200 sporozoites are transferred through a single mosquito bite (Itoe et al., 2014; Gomes et al., 2016). The sporozoites then find a capillary in order to enter circulation – a process that can take upto 3 h (Yamauchi et al., 2007; Aly et al., 2009; Ejigiri and Sinnis, 2009). During this process, the sporozoite has to traverse through the cells of the dermis which mean that several host–parasite interactions begin the moment parasite enters its human host (Ejigiri and Sinnis, 2009; Ménard et al., 2013).

The sporozoites also encounter the human immune response as soon as they enter the dermis, however, sporozoite survival is facilitated by its potent solvent of mosquito saliva, antihistamines, vasodilators (tachykinin), anticoagulants (thrombin), platelet aggregation inhibitors, and immunomodulators. It has been shown that most sporozoites once inoculated, travel via the blood stream to reach the liver, however, a small proportion may also travel via the lymphatic system by recruiting macrophages (Amino et al., 2006; Frevert et al., 2006; Aly et al., 2009). Although this has not been demonstrated in humans yet, both mouse and avian *Plasmodium* parasites have been found within macrophages. If true then this would be the first step toward host cell remodeling and immunomodulation that is performed by the parasite and a very important target for therapeutic intervention.

**FIGURE 1:**
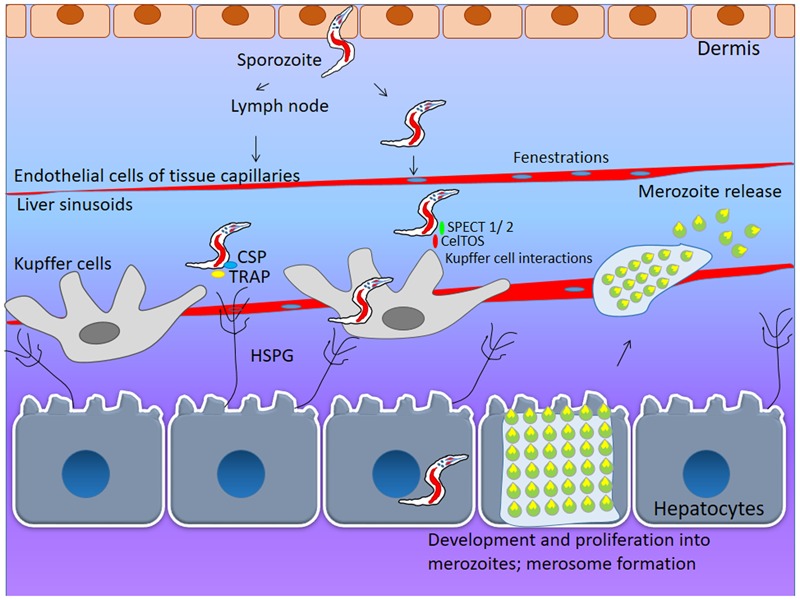
**First line of interaction: Skin and liver Hepatocyte Invasion.** During initial stages of *Plasmodium*-host interaction, the mosquito vector (female *Anopheles*) injects 20–200 sporozoites under the dermis from where it travels into circulation via lymph nodes. These sporozoites enter the liver sinusoidal space by using fenestrations in tissue capillaries. To invade the human hepatocyte, the sporozoite needs to interact with surface lining of heparan sulfate proteoglycans (HSPG). The sporozoite surface proteins- circumsporozoite protein (CSP) and thrombospondin-related adhesive protein (TRAP) help in invasion of liver cells by interacting with surface heparan sulfate molecules. During the cell transversal, parasite encoded transversal proteins viz: SPECT-1, SPECT-2, CelTOS facilitate sporozoite invasion of hepatocytes. Once sporozoite enter the hepatocyte it undergoes schizogonic proliferation and eventually the rupture of hepatic schizonts yeild several blood stage merozoites.

Once the parasite reaches the liver, it must invade and multiply within hepatocytes. When the sporozoite reaches the liver sinusoids- specialized blood vessels with fenestrated endothelium, they encounter liver resident macrophages known as the Kupffer cells (Lindner et al., 2012). The glycosylated scavenger receptor CD68 has been shown to mediate interaction with the sporozoite (Mota et al., 2001). Sporozoite specific proteins such as SPECT1, 2/Perforin like protein (PLP) and CelTOS are thought to mediate hepatocyte invasion by the parasite via Kupffer cells. This has been demonstrated through SPECT 1, 2 KO parasite lines that are unable to establish liver stage infections (Ejigiri and Sinnis, 2009). The surface of the human hepatocyte is lined with heparan sulfate proteoglycans (HSPG) of unknown identity (Yuda and Ishino, 2004). As soon as the parasite encounters heparan sulfate, it switches from migratory to invasive mode (Ejigiri and Sinnis, 2009). Another surface glycoprotein CD81 and its co-receptor SR-B1 have been shown to be essential for sporozoite invasion of hepatocytes (Yalaoui et al., 2008). It has been found that monoclonal antibodies against CD81, but not SR-B1, completely blocks the sporozoite invasion of hepatocytes (Foquet et al., 2015). The sporozoite form of the parasite that invades the liver, expresses circumsporozoite protein (CSP) and Thrombospondin-related adhesive protein (TRAP) on its surface (**Figure 1**) (Kappe et al., 2004; Vaughan et al., 2008; Ejigiri and Sinnis, 2009). These proteins interact with the heparan sulfate molecules to latch on to hepatocytes prior to invasion. Inspite of having a series of complex host–parasite interactions, this journey from the skin to the hepatocytes occurs within a few minutes. HSPGs are present on the basolateral membrane of the hepatocytes and treatment with heparitinase, which digests the HS moiety, has been shown to disrupt the binding of the CSP to hepatocytes (Vaughan et al., 2008). The normal physiological function of HSPG is the binding of chylomicron remnants (Zeng et al., 1998). It has been shown that the parasite and chylomicrons compete for HSPG binding in hepatocytes (Sinnis et al., 1996). Perhaps the absence of HSPG on the cells of dermis might explain non-invasion of the parasite in the skin.

The circumsporozoite protein (CSP) of *P. falciparum* is in fact, the antigenic component of RTS,S/AS01- the most successful subunit malaria vaccine (Mata et al., 2013). In this preparation, CSP is conjugated to the Hepatitis B surface antigen and is expected to prevent sporozoite invasion into hepatocytes. Phase 3 clinical trials indicated that the protection provided by RTS,S/AS01 was modest and not long lasting (Mata et al., 2013). This was a 3 years phase III efficacy study involving 8922 children (5–17 months at enrollment) and 6537 infants (6–12 weeks). This study demonstrated a partial 36.3% efficacy against clinical malaria in children and 25.9% in infants heralding a new era of hope that an efficacious malaria vaccine was possible to produce and utilize for mass immunizations (RTS, S Clinical Trials Partnership, 2015; White et al., 2015). As a result, even if the RTS,S/AS01 vaccine did not show 100% long lasting sterile protection against malaria, it might still have utility as a malaria control tool, in combination with other measures such as vector control and the use of insecticide-treated bednets. However, the quest for a malaria vaccine that will provide long-lasting sterile protection is still ongoing and the need for molecular understanding of host–parasite interactions in human clinical malaria is more than ever.

Whole organism vaccines remain one of the most powerful vaccination strategies against infectious diseases since they target an array of host–parasite interactions at the same time and eliminate the necessity of identifying specific vaccine candidates. In case of *P. falciparum*, the attenuated sporozoite has been recognized as a very effective vaccine candidate that is capable of generating long term sterile protection. However, in the initial phases of its development, the only way to deliver the sporozoite into human circulation was through the bite of the Anopheles vector, making it an effective, but impractical vaccine candidate (Richie et al., 2015). In a seminal advance, a whole organism vaccine using “attenuated, aseptic, purified, and cryopreserved PfSPZ” has been developed which allows for the possibility to develop a whole organism vaccine that can be mass produced, can be safe and effective, and can be stored and transported (Seder et al., 2013). This whole organism vaccine has been shown to be far more successful in achieving long-term sterile protection in controlled human malaria infection (CHMI) trials, than any subunit vaccine ever used. CHMI is a strategy to test interventions against malaria wherein a select population of consenting participants are inoculated with Pf sporozoites to induce malaria infection, either by direct venous injection of sporozoites or via mosquito bites (Engwerda et al., 2012). One of the major lacunae with the malaria subunit vaccines has been their inability to generate long term sterile protection. The PfSpz vaccine appears to have overcome this challenge, at least in the context of challenge in CHMI settings (Richie et al., 2015). RAS were demonstrated to confer immune protection to about 64% of vaccinated individuals against homologous challenge and to 83% of vaccinated participants in a subsequent challenge with a single heterologous strain in a CHMI study (Lyke et al., 2017). In the most recent and largest safety and efficacy study carried out as a randomized double blind trial in Mali, radiation attenuated PfSpz vaccines were delivered via direct venous inoculation to about 93 adult participants. About 66% of the vaccinated group developed naturally acquired malaria infections while about 34% were protected. This indicates that in the field-setting, there are many more aspects to be assessed toward developing an effective vaccine (Sissoko et al., 2017). Two additional strategies for the development of PfSpz vaccines have been tried successfully, namely, the use of a chemoattenuated PfSpz vaccine and PfSpz-GA1 (genetically attenuated) (Richie et al., 2015). The strategy for PfSpz chemoattenuation is to inoculate infectious sporozoites into CHMI participants who are under chemoprophylaxis with chloroquine, an approach termed as C-Vac. Recently reported CHMI trial with the C-Vac vaccination approach demonstrated that chemoattenuated parasites protected about 67% participants from an infectious PfSpz challenge in a dose dependent manner (Mordmüller et al., 2017). Immune sera from protected individuals recognized 22 proteins out of an array of 7455 *Plasmodium* peptides indicating that these 22 proteins constitute the most important subset of host interactors in the sporozoite stage. However, the dose, immunization regimen and time between intervals for all the PfSpz vaccination strategies need to be refined in order to achieve a truly efficacious malaria vaccine. The PfSpz-GA1 is a knock out parasite in which two proteins, *Pfb9* and *Pfslar*p, that are essential for liver stage development are deleted (van Schaijk et al., 2014). The deletion of these two genes allows liver infection of the parasite but has been shown to completely abort subsequent development in the liver. All the three different types of PfSpz vaccines have different mechanisms and stages at which their growth in the liver is arrested and are likely to have different efficacies (Richie et al., 2015). However, the PfSpz vaccines highlight the importance and effectiveness of targeting multiple host–parasite interactions rather than a few at a time. Since subunit vaccines are cheaper and easier to produce, store and transport, the findings from these whole sporozoite vaccine approaches can inform the development of new multi-subunit vaccines based on the host–parasite interactions found to be involved in generation of immunity.

Within the liver, the parasite replicates and emerges as merozoites- infective forms that commence the asexual blood stages of the malaria parasite (Gomes-Santos et al., 2011). Most of our understanding of replication within hepatocytes comes from animal models of the disease which show that host phospholipids are essential during early replication of the parasite post hepatocyte invasion (Itoe et al., 2014). While animal models of malaria and the parasites that infect them might use similar overall strategies, the molecular details might differ from human –parasite interactions. Therefore, molecular mechanisms of animal malaria models need to be validated for human malaria interactions.

After replication and maturation in the human liver which may take 10–12 days, *P. falciparum* merozoites are released into the circulation as merozoites that preferentially invade mature human RBCs (Prudêncio et al., 2006). An *in vitro* system of co-cultured hepatocytes and stromal cells on a microchip has been shown to support the growth of *P. falciparum* and *P. vivax* liver stages (March et al., 2013) which will possibly be instrumental in discovering human liver- parasite interactions that will have greater relevance to the design of novel therapeutic strategies against human malaria.

### Interactions in the Blood Stages: Invasion and Cytoadherence

**FIGURE 2:**
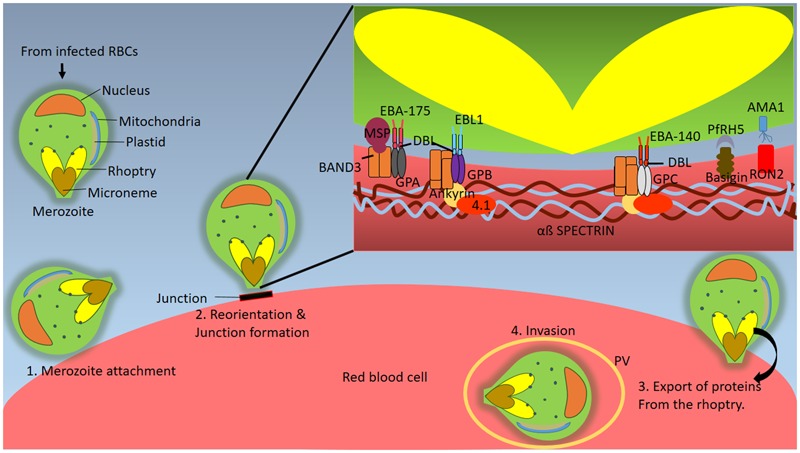
**Interactions in the Blood Stages: Invasion.** Subsequent to eruption of hepatic schizonts, several merozoites are released into the blood stream. Merozoites are well-defined pear shaped blood stage form of the parasite which have characteristic conserved organelles known as rhoptries and micronemes (Step 1) Merozoite initially attaches to the RBC surface which is followed by its (Step 2) reorientation and formation of a moving junction on RBC surface through its apical end. The moving junction acts as a connector between parasite and host cytoskeleton. During this process various molecular interactions take place. Band 3 gets phosphorylated leading to a decrease in its affinity for membrane cytoskeletal elements ankyrin and spectrin, resulting in modulation of host cytoskeleton and formation of parasitophorous vacuole within which the invading merozoite resides. Rhoptry neck protein (RON2) interacts with the hydrophobic pocket of Apical membrane antigen 1 (AMA1) triggering the formation of moving junction. The parasite ligands EBA-175, EBA-140 and EBL-1 are members of the DBL-EBP family and bind to glycophorins via DBL domain. PfRh5 is a ligand for the host receptor Basigin. PfRH5 binds to via its protein core instead of the glycans. (Step 3). This is followed by export of parasite encoded proteins into the RBC compartment that allow invasion to occur. (Step 4) Finally the merozoite is inside the RBCs and resides within the parasitophorous vacuole (PV) throughout the blood stages.

*Plasmodium* species belong to the phylum Apicomplexa. This nomenclature is based on the presence of two types of unique organelles within the parasite, the apicoplast (non-photosynthetic plastid) and the apical complex structure. An important characteristic of RBC-invasive merozoites (small, polarized pear-shaped cells) that emerge from the liver and at the end of every life cycle in the RBCs, is the presence of specialized subcellular structures known as rhoptries and micronemes (**Figure 2**). Merozoites can invade mature human RBCs using two independent mechanisms-one by utilizing sialic acid (SA) residues on host receptors and one independent of SA (Baldwin et al., 2014). Parasite-encoded molecules involved in the SA dependent pathway are the Erythrocyte Binding Ligands (EBL) family of proteins. The major sialylated proteins on the RBC surface are the glycophorins A, B, and C that have been shown to bind EBA-175, EBL-1, EBA-140, respectively (Gaur and Chitnis, 2011; Li et al., 2012; Lin et al., 2012). The EBL proteins are stored in micronemes which are apical organelles involved in the invasion process. Among the PfRH class of proteins, PfRH1 is the only member that binds SA residues.

PfRH2, PfRH4, PfRH5, and MSP (merozoite surface protein) are components of the SA-independent pathway (Ord et al., 2012; Baldwin et al., 2014). The merozoite surface is lined with large complexes consisting of MSP and processed peripheral fragments MSPDBL-1 and 2 that have been shown to be essential for invasion into human RBCs (Lin et al., 2014; Paul et al., 2015). These complexes interact with non- glycosylated regions of Band 3, the anion transport protein present in the RBC membrane (Paul et al., 2015). At the initial stages of invasion into the mature human RBC, Band 3 gets phosphorylated leading to its dissociation from membrane cytoskeletal elements ankyrin and spectrin, resulting in modulation of host cytoskeleton and formation of parasitophorous vacuole within which the invading merozoite resides (Fernandez-Pol et al., 2013). As soon as the merozoite has entered into the erythrocyte, it releases a phosphatase called Pfshelph2 which dephosphorylates Band 3 using a divalent metal cation dependent catalytic mechanism (Fernandez-Pol et al., 2013). This results in the re-sealing and restoration of the erythrocyte cytoskeleton.

While such well-orchestrated molecular events help the parasite efficiently establish infection, they also provide several points for therapeutic targeting. MSP-1 is one of the proteins that is being explored as a vaccine candidate not only for the prevention of *P. falciparum* but also for *P. vivax* infections (Hill, 2011; Dantzler et al., 2015). MSP1 based vaccines have been relatively less explored as vaccine candidates. The only phase II trial with a pure MSP1-based vaccine conducted in Kenyan children showed no significant protection from clinical malaria (Ogutu et al., 2009). However, significant protection was observed in a phase IIa trial where adults were immunized with MSP-1 along with chimpanzee adenovirus 63 (ChAd63) followed by modified vaccinia virus Ankara (MVA) (Sheehy et al., 2012).

PfRH4 has been shown to bind the complement receptor 1 (CR1) and PfRh5 is a ligand for the host receptor Basigin (Gaur et al., 2004; Awandare et al., 2011). PfRH5 binds to the protein core of Basigin and removal of glycans from Basigin does not affect the binding (Crosnier et al., 2011). Interestingly, PfRH5 has been shown to elicit neutralizing antibodies in *Aotus* monkeys that provides protection against heterologous infection with variable strains (Douglas et al., 2015). In humans, detectable levels of anti-PfRH5 have been found to have invasion inhibitory functions *in vitro* (Patel et al., 2013). PfRH5 is known to complex with *P. falciparum* RH5-interacting protein (PfRipr) and cysteine-rich protective antigen (CyRPA) on the merozoite surface. This complex is known to be essential for invasion, wherePfRH5 and PfRipr form the Basigin binding complex and CyRPA tethers them to the merozoite membrane via a GPI anchor and inhibitory antibodies against CyrPA have been found to block invasion (Reddy et al., 2015). An analysis of antibody subclasses revealed that increase in the levels of IgG3 subclass of antibodies against PfRH5 was strongly associated with reduced risk of malaria in human studies in children (Weaver et al., 2016). PfRH5 is an actively pursued anti-malarial vaccine candidate also due to the strain-transcending nature of anti-PfRH5 antibodies, which promises to provide neutralizing protection against a wide variety of parasite strains. In addition, in a novel approach, basigin has been proposed as a druggable target in malaria infections. Zenonos et al. (2015) have developed a high-affinity recombinant chimeric anti-basigin-1 antibody (Ab-1) against human basigin and demonstrated that Ab-1 is effective against several parasite lines. In addition, it is successful in clearance of blood stage parasites in mouse models at the same time, without any toxic side effects.

Several other parasite ligands such as EBA-175, EBA-140, and EBL-1 have been shown to interact with the Glycophorins A, C, and B, respectively, and are thought to be necessary for invasion. However, it is interesting to note that parasite knockouts of all the above ligands with the exception of PfRH5, show no effect on invasion indicating redundancy in sialic acid dependent invasion pathways (Kobayashi et al., 2013). The human ortholog of basigin has been shown to have a 10-fold higher affinity for PfRH5 compared to non-human basigins (Crosnier et al., 2011; Wanaguru et al., 2013). Since Basigin is relatively well conserved this suggests that only a small number of amino acid residues might be drug development and many of these are being pursued as potential candidates, as described above. Rodent malaria parasites do not have any known homologs to RH5 or “RH5-like” antigens and therefore, further characterization of PfRH5 as a vaccine candidate cannot be carried out in rodent animal models. Therefore, the only alternative for PfRH5 characterization to study *in vivo* RH5 efficacy before conducting human clinical trials are the non-human primate (NHP) models. Recent efficacy studies on PfRH5 in NHP models show that there is a causal link between invasion inhibition and protection against clinical malaria. In addition, the RH5 vaccine demonstrated heterologous protection when administered using a viral vector platform. The PfRH5 therefore is being pursued as one of the most promising malaria vaccine candidates (Douglas et al., 2015).

In addition to the above, interaction between apical membrane antigen (AMA1) and rhoptry neck protein (RON) has been shown to be important for triggering junction formation and thus invasion (**Figure 2**) (Srinivasan et al., 2011). AMA1 is localized in the micronemes and is transported to the parasite surface after the invasion has started whereas RON is secreted from the rhoptries near the RBC membrane and inserts itself there. RON binds to a hydrophobic pocket in AMA1 which is comprised of two PAN domains (Srinivasan et al., 2011). The functional implication of AMA1, which is conserved in Apicomplexan, in the invasion is derived from the study in which AMA1 knockout *Toxoplasma gondii* cannot invade the RBC (Mital et al., 2005). AMA1 has been explored as a vaccine target, since anti-AMA1 antibody titres have been shown to be associated with naturally acquired protection against malaria (Thomas et al., 1994; Udhayakumar et al., 2001). Immunization with AMA1 has been proven to provide protection against malaria infections in mice as well as monkeys (Deans et al., 1988; Narum et al., 2000). The AMA1 vaccine in humans, viz, FMP2.1/AS02_A_ is a recombinantly expressed protein, FMP2.1 which is prepared in an adjuvant system AS02_A_ (Narum et al., 2000)_._ Phase I clinical trial with FMP2.1/AS02_A_ was carried out in Malian children (*n* = 10) who were divided into three random groups, which received three different doses of the vaccine and safety, reactogenicity and immunogenicity studies were carried out. The vaccine was found to be devoid of toxic effects although local pain and swelling were observed. Antibody titres measured using ELISA showed at least a 100-fold increase in the anti-AMA1 antibody levels (Thera et al., 2010) compared to baseline levels, which were maintained for upto 1 year after the immunization. A randomized, double blinded phase II trial that was subsequently conducted involved 400 Malian children who were administered either 50 μg FMP2.1 in 0.5 ml AS02_A_ or 1 ml of purified chick-embryo rabies vaccine (Thera et al., 2010). These children were then followed up for 6 months for a primary endpoint of clinical malaria (fever with a peripheral parasite density of at least 2500 parasites per cubic mm of blood). The safety and immunogenicity were found to be similar as in phase I trials. The efficacy of this AMA-1 vaccine was not found to be significant with respect to the primary endpoint of clinical malaria. However, the vaccine showed strain-specific protection (64.3%) against parasites harboring a similar AMA-1 sequence as the vaccine, which was 3D7 derived (Thera et al., 2011). Therefore, although AMA-1 is immunogenic, and safe for use in humans, a major challenge in its use as a vaccine candidate is the strain specific immunity generated by it and its highly polymorphic nature. If these issues could be addressed, perhaps, AMA-1 could be pursued as a vaccine candidate against malaria.

Merozoite invasion also additional interactions such as with CD55, a surface receptor RBC invasion which is important for proper attachment (Egan et al., 2015). Parasite invasion is found to be significantly reduced in CD55 knockdowns. CD55null cells were also resistant to invasion (Egan et al., 2015). However, this has only been shown *in vitro* in cultured parasites and this needs to be investigated further.

*Plasmodium vivax*, the second most prevalent human malaria parasite in the world has a slightly different biology as compared to *P*. *falciparum*. *P. vivax* is known to use the Duffy antigen receptor for chemokines (DARC) which is a host receptor that has been shown to be important for *P. vivax* invasion of RBCs (Adams et al., 1990). DARC negative individuals are resistant to infection, thereby confirming that DARC interaction with Duffy binding protein is essential for invasion (Langhi and Bordin, 2006). A number of studies have shown that this interaction when inhibited with antibodies can block parasite invasion (Langhorne and Duffy, 2016).

Another process that has been considered for therapeutic intervention in the parasite, is egress, which involves the exit of the merozoite from the erythrocyte. It is a highly regulated process in which both the PVM and erythrocyte membrane (EM) degenerate and this process involves parasite encoded proteses such as falcipain-2 (cysteine proteases), plasmepsins (aspartic proteases) and a family of putative papain like proteases called serine repeat antigen SERA 1-9 (Blackman, 2008). Falcipain and plasmepsins act in the hemoglobin digestion pathway and are also involved in the digestion of the cytoskeletal proteins such as spectrin, ankyrin and 4.1 (Blackman, 2008). The specific function of SERA is not known but it is thought to be important for the EM rupture (Blackman, 2008). SERA5, a merozoite egress protease which has been shown to be an essential gene in *P. falciparum*, has been proposed as a drug target (Miller et al., 2003). Inhibition of proteolytic activation of SERA 5 has been shown to be inhibitory to merozoite egress (Alam and Chauhan, 2012). Another recently identified parasite antigen, *P. falciparum* schizont egress antigen- 1 localized to the PVM has been found to be important to merozoite egress. Antibodies against PfSEA-1 have been shown to arrest merozoite egress from infected RBCs and PfSEA-1 deletion caused a replication defect (Raj et al., 2014). In addition the secretion of proteins from the apical organelles in the merozoite is regulated by intracellular potassium concentrations (Cruz et al., 2012). With the exposure of merozoites to low potassium concentrations, parasite phospholipase C gets activated which results in the production of IP_3_ that releases calcium through IP_3_ receptors which in turn regulates an evolutionarily conserved phosphatase complex (Cruz et al., 2012). This signal transduction is a significant process which finally results in the release of microneme proteins that mediate sialic acid dependent invasion and can provide additional targets toward drug development.

Understanding of molecular mechanisms of host cell invasion by the malaria parasite, be it hepatocyte invasion or RBC invasion, is important since invasion processes in general, and those specific to *Plasmodium* species, involve several receptor-ligand interactions as well as specific enzymes. Both these groups of proteins are amenable to the development of blocking antibodies and small molecule inhibitors. The malaria parasite has proven to be hard to vanquish through immunization as well as drug-based therapeutics due to two major reasons- (a) The immunology of malaria infections is complex and we do not have a precise understanding of how host immune responses are modulated during parasite infections and the genetic, environmental and host specific factors that govern this are still in early stages of discovery; (b) The parasite itself has a tendency to rapidly develop resistance to drugs in malaria endemic areas. The mechanisms of drug action and the development of resistance are uncovered in the wake of the emergence of resistant parasites. In the light of these circumstances, it is essential to develop a “multi-pronged” approach to limiting parasite growth possibly by combining vaccination strategies with drugs targeting several molecules at the same time.

### Post-invasion Interactions of the Infected RBC: Cytoadherence

Following invasion, the merozoite must establish its life cycle in the mature human RBC (erythrocyte) which is devoid of any organelles. When the merozoite enters the RBC, it encapsulates itself within a parasitophorous vacuole within which it grows, replicates and communicates with its extracellular environment (Spielmann et al., 2012; Mantel and Marti, 2014). In order to do so, *P. falciparum* parasites have been shown to establish an intricate membranous network in the infected RBC cytosol, known as the Maurer’s clefts and tubulovesicular network, resembling eukaryotic secretory pathway.

**FIGURE 3:**
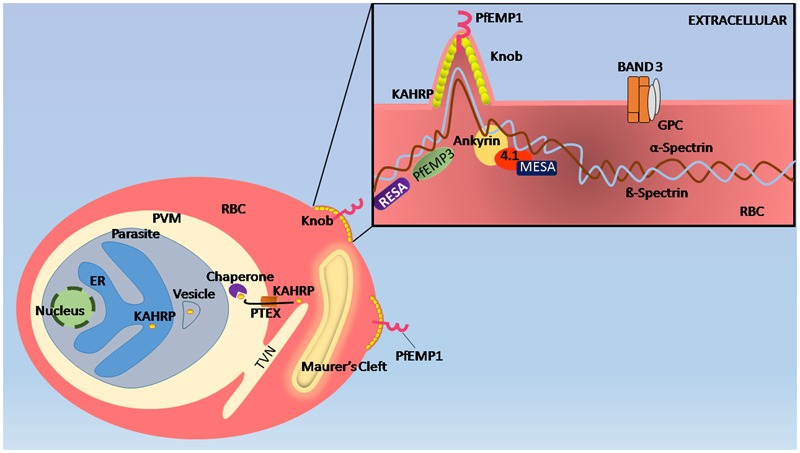
**Remodeling of erythrocyte membrane.** When merozoites enter the RBC, they are encapsulated within a parasitophorous vacuole (PV) within which they grow, replicate, and communicate with its extracellular environment. In order to do so, the parasite establishes an intricate membranous network in the infected RBC cytosol, known as the Maurer’s clefts and tubulovesicular network (TVN), which resembles the eukaryotic secretory pathway. In infected RBCs, RESA interacts with spectrin protein of the membrane cytoskeleton while *Plasmodium falciparum* knob-associated His-rich protein (KAHRP) interacts with spectrin under the infected RBC plasma membrane. PfEMP1 proteins that are responsible for cytoadhesion and immune evasion Host proteins 4.1R and spectrin helps in the placement of PfEMP 1 from Maurer’s cleft, are presented at knobs for which KAHRP is known to be crucial. PfEMP3 also binds to spectrin causing deformity in RBC membrane.

Through this network, the parasite secretes several parasite-encoded proteins into the extra-parasitic RBC compartment, the infected RBC plasma membrane as well as outside the infected RBC into the extracellular medium which in this case, is the circulation (Mantel and Marti, 2014; Rug et al., 2014). Toward this, the parasite has a novel set of signal sequences (such as the PEXEL/HT motif) and unique proteins and chaperones that aid in this process (Su et al., 1995; Melcher et al., 2010). Knowledge of the parasite protein export pathway and molecular interactions is very crucial toward understanding of well-known as well as potential virulence proteins encoded by the parasite. The most well-studied virulence proteins of the parasite are the members of the *var* gene family in *P. falciparum*. The var gene family consists of about 60 members in each strain of the parasite and encodes for the *P. falciparum* erythrocyte membrane proteins 1 (PfEMP1) family (Su et al., 1995). PfEMP1 proteins form the variant surface antigen family in the parasite. PfEMP1 are transmembrane proteins having a large hypervariable extracellular domain, a transmembrane domain and a shortconserved intracellular domain known as the acidic terminal segment (Su et al., 1995; Melcher et al., 2010; Weng et al., 2014). The large hypervariable extracellular domain enables the PfEMP1 to bind to various host receptors with differing affinities due to variation in their amino acid sequences (Weng et al., 2014). PfEMP1 proteins are a part of a large electron dense macromolecular complex on the infected RBC surface, known as knobs (Weng et al., 2014). PfEMP3 (*P. falciparum* Erythrocyte membrane protein 3), RESA (Ring- infected erythrocyte surface antigen), MESA (Mature-parasite infected ESA), and KAHRP (Knob associated Histidine-rich protein) are the structural components of knobs (**Figure 3**) (Weng et al., 2014). Several parasite-encoded proteins have been shown to be important in the formation of the knob such as the PHIST (Plasmodium Helical Interspersed Subtelomeric) domain protein (PFD1170c) and an Hsp40-like DNAJ Type IV protein (PF10_0381) as well as in the maintenance of the knob structure such as knob-associated Hsp40 (Acharya et al., 2012). Other parasite-encoded proteins form a structural part of the knob, such as PfEMP3 which is important in PfEMP1 trafficking, the high molecular weight variable surface antigen SURFIN, the Pf332 antigen- also a large protein of 2.5 MDa, and the PHIST domain protein PFE1605w (LyMP) (Hinterberg et al., 1994; Winter et al., 2005; Tarr et al., 2014). Surface cytoadherence is also known to be modified by proteins that are not knob-components, such as PFE1605w, which modifies cytoadherent characteristics of the infected RBC without influencing PfEMP1 expression or its structure (Oberli et al., 2014; Tarr et al., 2014). Details of interactions that have been documented to be important in knob formation are listed in **Table 1**.

**Table 1:** List of *Plasmodium* proteins that interact with host and *Plasmodium* proteins and their functions.

S.No.	Plasmodium protein	Interacting protein		Function	Reference
		Host	Parasite		
(1)	Skeleton binding protein 1 (SBP),	(1) 4.1R (2) Spectrin		Placement of PfEMP 1 from MC (Maurer’s cleft) to parasite-infected cell	Kats et al., 2015.
(2)	CD55	Not known	Not known	RBC invasion by merozoite	Egan et al., 2015
(3)	PHIST		PfEMP1 (ATS)	^∗^	Oberli et al., 2014
(4)	RESA	β spectrin		^∗^	Weng et al., 2014
(5)	MESA	Membrane domain of 4.1R		It is significant for the parasite growth in erythrocytes.	Weng et al., 2014
(6)	PfEMP1	(1) Directly with Spectrin (2) Ankyrin (3) 4.1R	Indirectly via KAHRP	Lead to its attachment to the membrane of infected RBC	Weng et al., 2014
(7)	MSP1 (fragments)	bind to the S100- protein (a proinflammatory cytokine)		Inhibition of NF-kb activation and blocking the inflammatory response	Waisberg et al., 2012
(8)	AMA1		RON2	Triggers the junction formation and lead to the invasion of parasite in RBCs.	Srinivasan et al., 2011
(9)	ETRAM	(1) Host apolipoproteins	Parasite heat shock protein	Lead to presentation of PfEMP 1 to infected RBC	Vignali et al., 2008
(10)	CD81	Not known	Not known	Hepatocyte invasion	Yalaoui et al., 2008
(11)	PfEMP3	(1) Appears on cytoplasmic surface of the host cell membrane (2) 60-residue fragment (FIa1, residues 38–97) of PfEMP3 binds to spectrin		(1) PfEMP3 junctions of the membrane skeletal network (2) PfEMP3 fragment causes extensive reduction in shear resistance of the cell	Pei et al., 2007
(12)	CD68	Not known	Not known	Sporozoite invasion	Mota et al., 2001

*^∗^Function unknown.*

PfEMP1 proteins interact with a wide variety of known (and possibly several unknown) host receptors present on the membranes of endothelial cells (Duffy et al., 2016). The N- terminal region of PfEMP1 consists of the Duffy binding- like domain (DBL) and a cysteine-rich interdomain region (CIDR) (Duffy et al., 2016). The high degree of sequence diversity in PfEMP1 is concentrated in the CIDR and DBL extracellular domains. The DBL domains are classified into several subclasses based on relative sequence similarities: DBL domains α, β, γ, δ, 𝜀, X and CIDR domains α, β, and γ. Known binding preferences of the different DBL domains are listed in **Table 2**.

**Table 2:** Summary of host receptors for PfEMP1 adhesion.

Subclass of PfEMP1 binding domain	Host receptor	Effect on the host	Reference
CIDR-α and DBL-β	CD36 and ICAM-1	Support adhesion to endothelia and platelet mediated clumping of infected RBCs	Arman et al., 2013
DBL X and DBL-𝜀	Placental receptor Chondroitin sulfate A	Placental sequestration of infected erythrocytes	Mens et al., 2010
Not known	Receptors on surface of immune cells	May modulate function of immune cells and reduce immune response against parasite.	Rowe et al., 2009
DBL-α1 and DBL-α	Complement receptor 1(CR1), A and B blood group antigens and heparin sulfate like molecule	Rosetting; binding of infected erythrocytes to uninfected erythrocytes	Rowe et al., 2009
CIDR-α and DBL-2δ	VCAM-1, PECAM-1, NCAM-1, P-selectin, E-selectin	Modulates endothelial properties such as permeability, apoptosis, and inflammatory response	Chakravorty et al., 2008

Binding of infected erythrocytes to human cell surfaces allows IE to sequester in deep vascular beds and avoids clearance of parasites by spleen. Cytoadherence of infected erythrocytes to normal RBCs makes them readily available for invasion by merozoites. Interestingly, this interaction is not inert and cytoadherence of the infected RBC to endothelial cell receptors activates downstream signaling pathways altering its properties- another example of host cell modulation by the parasite (Duffy et al., 2016). Although much of the research focus in past years has been on studying the structure and function of PfEMP1 proteins, there is increasing understanding that PfEMP1 alone does not determine the host cell binding characteristics of the infected RBC, and that there are many other parasite and host factors that mediate this process.

PfEMP1 is known to bind KAHRP which in turn interacts with the host spectrin, ankyrin and Band 4.1R (Weng et al., 2014). Some of the interactions of PfEMP1 with host or parasite derived proteins are listed in **Table 2**. KAHRP interaction with knob proteins has been shown to be essential in Knob assembly (Weng et al., 2014).

In recent years the Renin-Angiotensin system (RAS) has been implicated to be involved in intra-host parasite development and polymorphisms in the genes in the RAS pathway have been associated with protection against severe malaria (Silva et al., 2016).

Understanding direct host–parasite interactions have had powerful applications in development of vaccination strategies. VAR2CSA, which is the most highly conserved PfEMP1 across different parasite strains, is well known for its role in the placental malaria, which it mediates by the sequestration of parasites in placenta (Clausen et al., 2012). It interacts with the chondroitin sulfate A (CSA) on the syncytiotrophoblast cells on the placenta, through its DBL domains leading to parasite sequestration in the placenta of the developing fetus followed by localized inflammatory responses and placental malaria. Placental malaria can have deleterious consequences for the developing fetus such as preterm deliveries and low birth weight neonates. The disruption of this interaction is an excellent target toward therapeutic intervention and anti-VAR2CSA vaccines are under clinical trials (Clausen et al., 2012). PAMVAC (Placental Malaria Vaccine) is a VAR2CSA based protein vaccine that is undergoing phase I clinical trials that are expected to conclude at the end of 2017. The vaccine consists of a small sub-unit of the VAR2CSA protein (ID1-ID2a) that is expected to elicit protective antibodies that will block the VAR2CSA-placental CSA interaction and thereby prevent parasite sequestration at the placenta. In animal models, recombinant PAMVAC has been shown to elicit antibodies that will inhibit binding of homologous parasites to CSA in an *in vitro* binding assay^1^. The results of this trial are much awaited.

### The Sexual Stages and Transmission Blocking Vaccines

During blood stage development, some rings develop into sexual stages known as gametocytes. Gametocytes, are taken up by the mosquito during a blood meal and subsequently develop in the mosquito midgut forming ookinetes (Barillas-Mury and Kumar, 2005). The ookinete then traverses the mosquito midgut from the basal to the luminal side, emerging into the midgut lumen and developing into an oocyst, the only extracellular form of the malaria parasite (Barillas-Mury and Kumar, 2005). All the entry, exit and traversal mechanisms in the mosquito midgut, lumen and the salivary glands involve a completely different set of direct and indirect molecular interactions between the parasite and its mosquito host. The ookinete, which has apical organelles specializing in host cell recognition and invasion, has been shown to be an effective target for transmission blocking vaccines (TBV) (Dessens et al., 1999). The zygote, which results from the fusion of the micro and macrogametes in the mosquito midgut, is also a target for the development of transmission blocking vaccines. The protein–protein interactions that occur specifically between the sexual stages of the parasite and the mosquito vector have been elegantly reviewed in Aly et al. (2009). From all the known interactions between *P. falciparum* sexual stages and the mosquito midgut, the proteins that are being actively pursued as candidates for transmission blocking vaccines are the *P. falciparum* proteins Pfs230, Pfs25/Pfs28, and Pfs48/45(Kapulu et al., 2015) and the mosquito midgut ligand alanyl aminopeptidase N1 (APN1). The surface of the *P. falciparum* zygote is lined with Pfs25 and Pfs28 line the *P. falciparum* zygote in the mosquito midgut and Pfs230 is present on the gametocyte surface within the human host (Kapulu et al., 2015). Pfs 48/45 are present on the surface of the gametocytes as well as the male and female gametes (van Dijk et al., 2001). TBV have recently come of age as clinical trials with the above antigen have begun in the earnest. A recent efficacy study for the assessment of the TBV potential of the above antigen clearly demonstrates that IgG obtained from vaccination with Pfs230 and Pfs25 were able to completely block *P. falciparum* transmission of both the laboratory strain NF54, as well as the field isolates, as demonstrated by standard membrane feeding assays (Goodman et al., 2011; Kapulu et al., 2015). In contrast, Pfs 48/45 was partially effective and APN1 did not show significant transmission blocking activity (TBA). TBA was measured as oocyst intensity and prevalence. These are very promising novel vaccination strategies that will be tried in the field in the future.

There exists another relatively less understood aspect of gametocyte development that can potentially be a point of therapeutic intervention. Prior to their uptake by the mosquito, the gametocytes undergo a long process of development within the human blood, lasting from 7 to 10 days within which they mature from early stage I gametocytes to mature stages IV and V gametocytes (Goodman et al., 2011; Kapulu et al., 2015). Only the mature stage gametocytes are found in peripheral circulation, suggesting that the process of gametocyte development from stages I through IV involve sequestration in some tissues, perhaps as a mechanism of immune-evasion (Goodman et al., 2011; Kapulu et al., 2015). Studies carried out on histological analysis of tissue sections from autopsy of deceased patients reveal the human bone marrow as a primary site of gametocyte development (Joice et al., 2014; Gardiner and Trenholme, 2015). However, the molecular mechanisms involved in gametocyte-sequestration is different from the cytoadherence that occurs in asexual blood stages since the gametocyte surface does not contain either PfEMP1 or KAHRP, two most important proteins of the cytoadhesive complex present on the surface of iRBCs (Gardiner and Trenholme, 2015). In fact, unlike the asexual blood stages, gametocytes are believed to develop in the extravascular spaces in the bone marrow (Farfour et al., 2012). The interactions between committed iRBCs and the cells of the bone marrow are only beginning to be understood and drugs or antibodies that target these interactions could be potential tools for controlling malaria transmission (Nilsson et al., 2015).

## Indirect Interaction

Although the malaria parasite, *P. falciparum* in particular, is skillful in evading the human immune system, it is the human immune responses during malaria infections that determine disease outcomes such as severe malaria. In recent years, several indirect interactions between the parasite and the human host have been uncovered that clearly modulate the severity of inflammatory responses directly or indirectly (D’Ombrain and Robinson, 2008). Most of the outcomes of host immune responses have been measured in terms of cytokine responses. Several cytokines such as IL10, TGF-ß, IL-17, IFN-γ, TNF-α, IL-6, IP-10, TNF-R2, TLR2, IL-8, IL-15, MCP-1, EOTAXIN, IL-5 are found to have altered expressions in different conditions of malaria (**Table 3**). The IFN-γ responses seem to be important in early control of *P. falciparum* infection and protection against severe malaria (Prakash et al., 2006). In fact, the peripheral levels of IFN-γ seem to reduce before onset of severe malaria symptoms in humans (Lelliott and Coban, 2016). IFN-γ has recently found to inhibit the liver stage sporozoite invasion and the mechanism seems to be similar to LC3 associated phagocytosis in which primarily LC3 attaches to the membrane of vacuole containing pathogen leading to the fusion of lysosome and resulting in phagocytosis (Lelliott and Coban, 2016). Interleukin IL-18 has been shown to have a protective role during malaria infection which it mediates through the IFN-γ production (Angulo and Fresno, 2002). Interleukin Il-10 switches the immune response from Th1 to Th2 mediated response (Angulo and Fresno, 2002). Human NK cells have also been found to be activated in the early stages of malaria infection. There seems to be a direct contact of infected RBCs with NK cells as evidenced by *in vitro* activation of NK cells resulting in IFN-γ production (Orago and Facer, 1991; Tsakonas et al., 2003). However, the role of NK cells in malaria infection is still under investigation. TGF-ß found to have dual, i.e., pro- as well as anti- inflammatory, roles (Angulo and Fresno, 2002). TNF-α has been found to reduce the invasion of *P. falciparum* into host RBCs (Cruz et al., 2016). TNF-α is hypothesized to bind to a putative ATP binding protein on the surface of the infected RBC which in turn increases the intracellular calcium concentrations (Cruz et al., 2016). The intracellular calcium rise due to TNF was found to decrease the expression of *P. falciparum* proliferating cell nuclear antigen-1 leading to a decrease in parasite proliferation within the host RBC (Cruz et al., 2016). Therefore, cytokines appear to have variable roles in malaria infections and a complex interplay and as-yet undiscovered mechanisms of action of these inflammation-mediators might influence disease outcomes.

**Table 3:** List of cytokines, eNOS, NO with altered expressions in different condition of malaria.

S.No.	Species	Cytokine UP(+) OR DOWN(-)	Comments	Reference
(1)	*P. vivax*	IL-17 + IFN-gamma + TGF-β **-**	Alterations of blood viscosity Reduction of viscosity by IL-17-possible immunomodulator	Scherer et al., 2016
(2)	*P. falciparum*	TNF-alpha + IL-6 +	Significantly higher in severe malaria	Mbengue et al., 2016
(3)	*P. falciparum*	IP-10 + TNF-R2 +	Significantly + in cerebral malaria	Jain et al., 2008
(4)	*P. falciparum*	IL-8 + IL-15 + MCP-1 + Eotaxin **-** IL-5 **-**	Poorly controlled inflammatory response determines a bad outcome	Dieye et al., 2016
(5)	*P. falciparum*	Endothelial nitric oxide synthase (eNOS) (Glu2983Asp substitution and “C-b-Asp” haplotype)	Protective effects against cerebral malaria and that the presence of Asp at position 298 may influence eNOS expression and NO production by the “C-b” haplotype.	Dhangadamajhi et al., 2009
(6)	*P. falciparum*	IL-10 **-**	Proinflammatory	Jason et al., 2001
(7)	*P. falciparum*	Nitric oxide (NO)	Increased NO production has been shown to be beneficial because of its antiparasitic and antidisease effect. Nitric oxide (NO) decreases erythropoiesis, and it is likely an important mediator of anemia of chronic disease	Anstey et al., 1999

A potentially direct interaction between the host immune system and *P. falciparum* seems to be mediated by the parasite homolog of mammalian macrophage migration inhibitory factor (MIF) (Miller et al., 2012). The mammalian MIF causes a proinflammatory response by inhibiting glucocorticoid-mediated downregulation of inflammation. MIF is highly conserved among different organisms and its presence in the parasite increases the effect of the mammalian MIF, i.e., increasing the proinflammatory response and possibly determining disease outcomes in the infected individual (Miller et al., 2012).

Parasite infected erythrocytes bind to human CD36 via PfEMP1 and phosphatidylserine (present in the membrane bilayer) and subsequently attach to the dendritic cells decreasing their ability to activate the T cell population (Urban et al., 1999; Robinson et al., 2003). Several soluble parasite ligands such as phosphate esters are known to bind host TLRs resulting in the activation of Yδ T cell. These activated immune cells produce large amounts of IFN-γ (Stevenson and Riley, 2004). These and several other interactions might modulate human immune responses against malaria and deeper molecular understanding of these interactions may lead to novel intervention strategies.

Parasite derived metabolites such as *P. falciparum* Glycosylphosphatidylinositols (GPIs) are known to cause pro-inflammatory cytokine production and GPI levels positively correlate with severe malaria (SM). GPI also acts as a ligand for the CD1d-restricted NK cell populations leading to activation of NK cells and secretion of IFN-γ. Anti-GPI antibody has been found to be effective in suppressing SM (De Souza et al., 2010).

## Future Directions

Although malaria is caused by *Plasmodium* infection of humans, it is a multi-factorial disease and malaria outcomes have been shown to depend on a wide variety of factors- from parasite species, strain, host immune responses, host as well as parasite metabolic pathways, genetic polymorphisms, to name a few. Until recently, most virulence research in malaria biology focused on the variant surface antigen family of the parasite which is important to parasite virulence but not the sole player in this complex disease. It is now well recognized that several host–parasite interactions, beginning just after infection at the human dermis, followed by the liver stages of the parasite, the blood stages of the parasite, as well as the indirect interactions of the host immune system and the parasite, need to be investigated in detail. Several efforts at vaccine design have been partially successful but none have lead to complete and long term protective immunity.

Most of the host–parasite interactions that have been examined up until now are interactions between protein effectors. However, recent data points toward novel roles performed by regulatory nucleic acids such as antisense and long non-coding RNA that have been shown to regulate both var gene expression and gametocyte development (Amit-Avraham et al., 2015; Broadbent et al., 2015). Although we need a better understanding of these processes in the parasite, these might be the therapeutic targets of the future.

The identification and targeting of the lowest common denominator of host parasite interactions in malaria, instead of a limited subset of proteins, might enable design of effective vaccines and therapeutic interventions against this elusive parasite.

## Author Contributions

PA conceived of the manuscript, wrote a major portion of the manuscript, coordinated and directed the team. MG carried out the literature survey, wrote a major portion of the manuscript, constructed the tables and the figures. PK edited and formatted the manuscript, contributed specific portions of the manuscript. He has also contributed to construction of tables. AM and KR edited and proofread the manuscript.

## Conflict of Interest Statement

The authors declare that the research was conducted in the absence of any commercial or financial relationships that could be construed as a potential conflict of interest.

## Footnotes

1 https://clinicaltrials.gov/ct2/show/record/NCT02647489?term=VAR2CSA&rank=1
